# Modeling and Experiments on Multilayered Barrier Coatings Containing Water-Sorbent Biopolymers

**DOI:** 10.1021/acs.iecr.5c04116

**Published:** 2025-11-25

**Authors:** Solomon Stavros Melides, Ian Williams, Joseph L. Keddie

**Affiliations:** School of Mathematics and Physics, 3660 University of Surrey, Guildford GU2 7XH, U.K.

## Abstract

Environmental regulations and consumer demand are driving the need for packaging materials to reduce or eliminate the use of petroleum-based plastics by replacing them with natural and degradable alternatives. Water flux through a barrier layer is governed by the differential in water activity between a high side (e.g., an aqueous solution) and a low side, such as humid air. We propose that a water-absorbent layer sandwiched between two barrier layers will act as a sponge and locally raise the water activity. It will thereby lower the activity differential across the first barrier layer and, hence, reduce the water flux through the multilayer for a set time period. We present a theoretical model that predicts the water flux through a multilayered structure of two or more barrier layers sandwiching hydrophilic layers that hold water according to an absorption isotherm. We use this model to evaluate the effects of layer thicknesses and distributions, the barrier permeability, the water-holding capacity, and initial water activity of the absorbent layers. We found that the water mass loss rate decreases when the thickness and water-holding capacity of the absorbent layer increase. The greatest reduction in the mass loss through a multilayer was achieved when the absorbent layer had an initial water activity of 0 (fully dried). The relative thicknesses of the barrier layers in the multilayer also have an impact on the water loss rate; thicker barrier layers on the low-activity side of the multilayer are the most effective in reducing the water flux. We have verified the model in an ideal system. We also investigated a multilayer structure of a waterborne emulsion polymer barrier coating and water-absorbing chitosan, which is a deacetylated form of chitin. Here, the chitosan layer offered little benefit in decreasing the water permeability because the layers were not thick enough and the permeability of the waterborne coating on its own is not sufficiently low. With our design concept for multilayer barriers containing absorbent layers and the underpinning theoretical model, we envisage materials systems with enhanced barrier properties while also using less petrochemically derived plastic.

## Graphical abstract

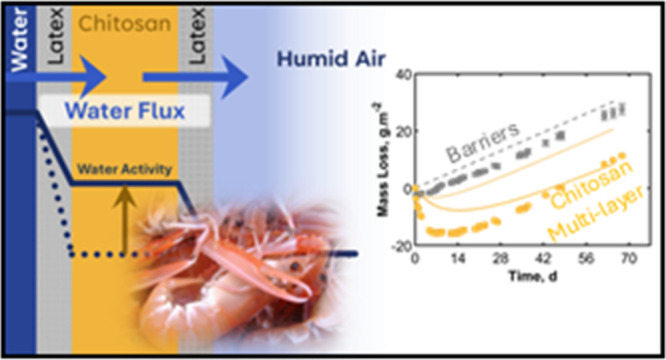

## 1 Introduction

The negative impacts of petrochemically derived plastics on our planet^1^ and human health^2^ are well documented.^3–5^ There are regulatory drivers^6^ as well as growing consumer pressure to reduce single-use plastic packaging and to replace it with sustainable alternatives. This presents a challenge to packaging manufacturers because petroleum-based plastics offer low permeability to liquids and gases in combination with the required mechanical properties.^4,7,8^ Recent research has investigated the used of bioderived feedstocks for packaging materials,^9^ which reduce the dependence on fossil fuels and can potentially lower the carbon footprint of manufacturing. Such biobased polymers, e.g. biopoly­(ethylene terephthalate), are chemically identical to the petroleum-based version, are not biodegradable, and can often have a higher carbon footprint.^10^ Alternatively, there has been interest in the packaging industry in using biopolymers that are biodegradable, but these materials typically do not offer the same barrier properties as conventional plastics.^4^ In recent years there has been growing interest in chitosan,^11^ a deacetylated chemical derivative of chitin. Chitin is the second most abundant naturally occurring biopolymer after cellulose. It is found in the cell walls of fungi as well as in the exoskeletons of shellfish and insects. Chitosan is a biodegradable copolymer of β­[1,4]-linked 2-acetamido-2-deoxy-d-glucopyranose and 2-amino-2-deoxy-d-glucopyranose,^12^ as is illustrated in Figure 1. Its production is relatively inexpensive, and chitin sources are relatively abundant, as they are a waste product from the seafood industry. Chitosan has been reported to have one of the lowest water permeabilities of all sustainable biobased materials.^13^ Hence, there is growing interest in using chitosan in packaging as a sustainable alternative to petroleum-derived plastics.

**Figure 1:**
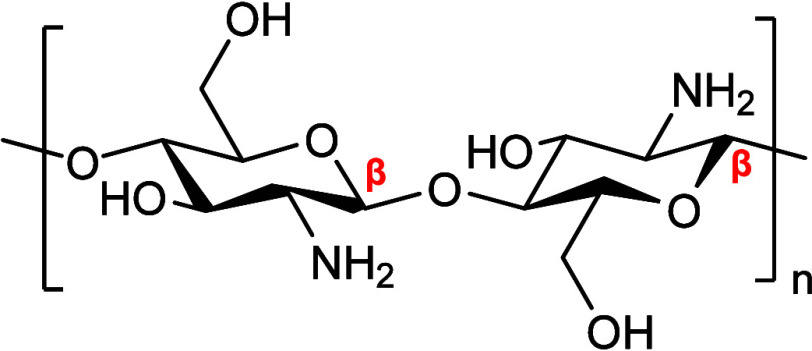
Molecular structure of the repeating group of chitosan. The image is redrawn from ref 12, which was published under a Creative Commons CC by License (https://creativecommons.org/licenses/by/4.0/). Copyright 2022. Guarnieri et al.

Even so, on their own, chitosan and other biobased materials seldomly have sufficient barrier properties for the long-term storage of liquids. The use of multilayered coatings is an attractive option. The flux of small molecules through a barrier layer is determined by the differential in activity from one side of the barrier to the other. By judiciously including a water-sorbing layer, which locally changes the gradient in activity in a multilayer structure, we theorize that the flux of water can be reduced for a period of time. Here, we propose using chitosan in combination with a conventional barrier coating. We present a new theoretical model to predict the barrier properties of multilayered coatings along with experimental results to verify the model. This new approach to building a barrier has the potential to reduce the use of fossil fuel-derived plastics while achieving acceptable properties.

## 2 Theoretical Basis

The effectiveness of a water barrier can be characterized by the flux of water from one side to the other, as is shown in Figure 2A. The flux, *J*, is driven by the gradient in potential of the partial pressure of water across the barrier. Typically, this is presented as the water activity (*a*_w_) which is a ratio of the partial pressure of water above a sample to a fully saturated equivalent at the same pressure and temperature. Specifically, *J* depends on the differential in water activity, Δ*a*_w_, on the two sides of the barrier as

$$J=\frac{D\cdot \rho \cdot \Delta a_{w}}{L}$$ 1

where *D* is the diffusion coefficient of water in the barrier, ρ is the solubility of water in the barrier, and *L* is the thickness of the barrier. Typically, the effectiveness of a barrier is increased either by decreasing its permeability, *p*, which is the product of *D* and ρ, or by increasing the barrier thickness, *L*. Often overlooked, however, is the idea that *J* can be slowed by reducing Δ*a*_w_.

**Figure 2:**
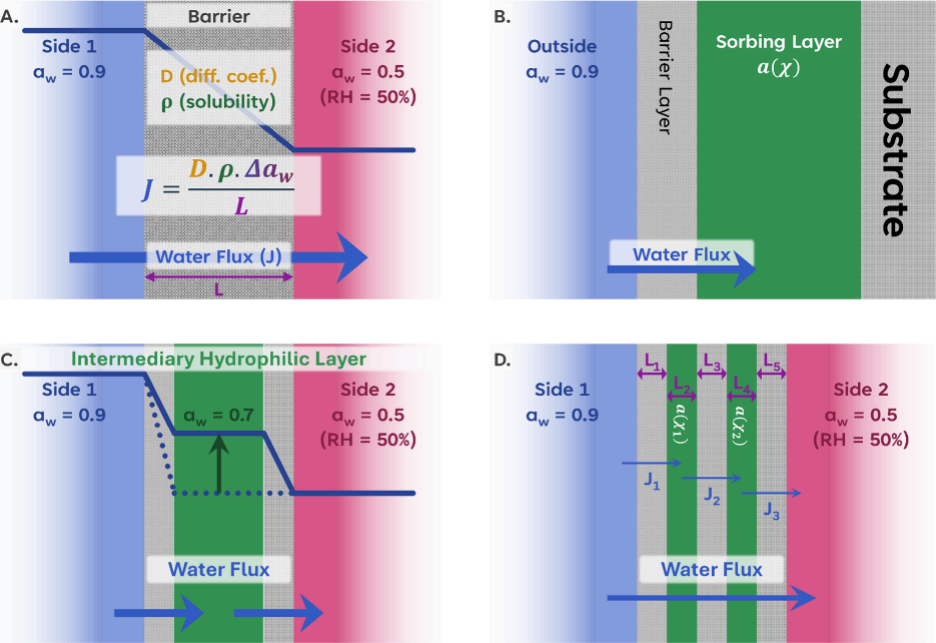
(A) Schematic diagram of the flux of water from a surrounding of high-water activity (0.9 *a*_w_, such as in an aqueous solution) through a barrier to a phase with a lower water activity (0.5 *a*_w_, such as in humid ambient air). (B) Schematic diagram of the bilayer structure used in the model of Baukh et al.,^14^ where water passes through a barrier into a sorbing layer. The diagram is redrawn from ref 15. (C) Redrawing of schematic of panel A with the insertion of a sorbing layer between two barrier layers. (D) Schematic representation of a five-layer design indicating the fluxes across each barrier.

Baukh et al.^14,15^ considered the effect of Δ*a*_w_ on the rates of water uptake in a bilayer coating system. Their bilayer consisted of an outer hydrophobic barrier coating on an inner hydrophilic water-sorbing layer coating an impermeable surface, as is represented Figure 2B. They developed a model that numerically considered only the permeability of the barrier coating (not its water sorption) and only the water sorption of the hydrophilic layer (not its water permeability). Baukh et al. modeled the water flux through the bilayer by dividing the differential in activity by a characteristic time (τ) that includes some of the parameters in eq 1. The water activity of the sorbing layer inside the bilayer (*a*_in_) varies with its water content as water diffuses across the barrier into it from the outside environment with an activity of *a*_out_. When water diffuses into the bilayer, the mole fraction of water content in the sorbing layer, χ, increases at a rate of:

$$\frac{\delta \chi }{\delta t}=\frac{a_{\mathrm{out}}-a_{\mathrm{in}}\left(\chi \right)}{\tau }$$ 2

where *t* is time and $\tau =\frac{\left(Ln_{\max}\right)}{AD\rho }$ with the unit area of *A*. The water molar fraction is calculated as $\chi =\frac{n_{S}}{n_{\max}}$ where *n*_S_ represents the number of water molecules in the sorbing layer (expressed in moles), and *n*_max_ is the maximum number of water molecules (in moles) that the sorbing layer can hold when saturated. The Baukh model makes three assumptions. One, it assumes the barrier layer absorbs negligible amounts of water, has a low diffusion coefficient and that the water activity is solely a function of the base coat water content. Two, it assumes water diffusing through the barrier layer will act like an ideal gas. Three, it assumes that there is an instantaneous redistribution of water in the sorbing layer. The water that migrates into the sorbing layer will raise its water content, which in turn will raise its water activity. The relationship between sorbed water and the resulting water activity is nonlinear and typically presented as a sorption isotherm. In the Baukh model, a sorption isotherm was used to relate the water content to the water activity of the sorbing layer. Baukh et al. considered linear, Type II and Type III isotherms. They found that with a Type III isotherm, the rate of water uptake in the sorbing layer was slow compared to when a simple linear isotherm was used. That is, when the water activity in the sorbing layer was raised, the driving force for the water flux across the barrier layer was reduced. This concept has inspired our present work.

To date, no predictive tool has been developed to describe the relationship between the water activity and the sorbed water content. The relationship must therefore be obtained experimentally and described using semiempirical equations. Typically, the water sorbed by the system (*m*_bulk_) is presented as a mass ratio of sorbed water (*m*_water_) to the mass of the fully dry sorbing material (*m*_dry_). In our simulations presented here, the Guggenheim–Anderson–de Boer (GAB) equation^16^ is used to express the sorption isotherm to obtain *m*_bulk_

$$m_{\mathrm{bulk}}=\frac{m_{\mathrm{water}}}{m_{\mathrm{dry}}}=\frac{m_{\mathrm{mono}}\cdot C\cdot K\cdot a_{w}}{\left(1-K\cdot a_{w}\right)\cdot \left(1-K\cdot a_{w}+C\cdot K\cdot a_{w}\right)}$$ 3

where *m*_mono_ is the dimensionless mass of water needed to be adsorbed to form a monolayer at the sample surface, and the *C* and *K* values are used as dimensionless fitting parameters. Chemically, the *C* value is described as being the difference in chemical potential between a water monolayer and the sorption layers above it, and the *K* value is linked to the difference in chemical potentials between pure water and the top layers (0 < *K* < 1). Figure 3 shows some examples of sorption isotherms obtained by using eq 3 with a range of parameters. Here, *m*_bulk_ is expressed conventionally as a percentage. Higher values of *m*_mono_ increase the magnitude of the sorption. The value of *C* alters the initial shape of the curve, with higher values causing a greater initial rise in the mass uptake at low water activity. As *K* approaches 1, the final values of the sorption isotherm are much greater. (The equation is not defined for *K* = 1). As can be seen in the figure, *m*_mono_ and *K* are of the greatest significance in determining the overall shape.

**Figure 3:**
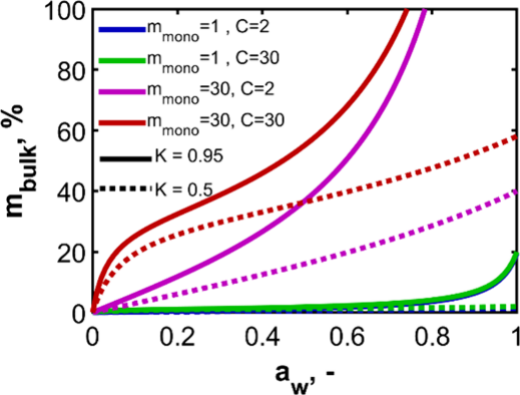
Effect of each GAB parameter on the shape of water sorption isotherms, which are obtained from eq 3, expressed as percentage. Solid lines are when *K* = 0.95, and dotted lines are when *K* = 0.5. The legend shows the values for the other two parameters.

We extended the Baukh model by allowing water transport from one side of a multilayer structure to the other (removing the impervious surface). To stop the water-sorbing layer from drying out we added a second barrier layer in the structure, as is illustrated in Figure 2C, thereby making a trilayer system. The function of the first barrier layer is to prevent ingress of water from the high activity side, and the function of the second barrier is to prevent egress of water from the sandwiched sorbing layer. Configuring a barrier such that there is an intermediary water-sorbing middle layer alters the local water activity and Δ*a*_w_ in eq 1. When the sorbing layer has the same initial activity as does the water activity outside the barrier, the differential in water activity across the final barrier layer is zero, and thus the overall flux is zero. When water is sorbed into an intermediate layer, the local water activity will rise. The differential between the intermediary layer and the surroundings will begin to increase until eventually the system is in equilibrium, with the activity in the sorbing layer taking a value intermediate between the two sides (see Figure 2C).

Table 1 lists some common biobased materials and their GAB values, as found in the literature. Values obtained from different research groups can vary greatly. Certain values (highlighted in bold italic font) vary from the understood physical constraints of the model (0 < *K* < 1, and *C* > 0)^17^ and thus highlight how the GAB values are often treated solely as fitting parameters. The effect of *K* on the slope of the isotherm when its value is <0.4 becomes small and the isotherm depends more strongly on *C*. When the *C* value is <2, the isotherm’s shape is not sigmoidal.^17,18^

**Table 1:** List of Common Biopolymers and Their GAB Values

**sample**	**temperature °C**	***m*_mono_** **g/g**	***C***	***K***	**authors**
**cellulose and paper**					
vegetable parchment paper	25	3.5	6.4	0.84	Rhim 2011 ^19^
kraft paper	25	3.0	9.9	0.87	Rhim 2011 ^19^
bleached kraft paper		4.1	9.6	0.67	Bedane et al. 2015 ^20^
solid bleached sulfate paperboard	25	3.2	8.2	0.85	Rhim 2010 ^19^
cellulose film		8.0	5.0	0.56	Bedane et al. 2015 ^20^
nanofibrillated cellulose		11.9	2.6	0.53	Bedane et al. 2015 ^20^
cotton liner		7.2	3.8	0.65	Bedane et al. 2015 ^20^
carboxymethyl cellulose	20	9.1	5.9	0.96	Bedane et al. 2015 ^20^
carboxymethyl cellulose film		7.2	3.5	0.62	Bedane et al. 2015 ^20^
**starch derivatives**					
maltodextrin Mc	30	23.3	6.3	***0.11*** ^a^	Saavedra-Leos et al. 2015 ^21^
maltodextrin (DE10)	30	5.3	8.6	0.70	Saavedra-Leos et al. 2015 ^21^
maltodextrin (DE20)	30	41.3	1.1	***0.32***	Saavedra-Leos et al. 2015 ^21^
maltodextrin (DE40)	30	4.1	11.6	0.95	Saavedra-Leos et al. 2015 ^21^
**other polysaccharides**					
agar	25	14.0	65.0	0.89	Rhim 2011 ^19^
alginate		124.0	***0.0*** ^a^	***1.31***	Xiao and Tong 2013 ^22^
carrageenan	20	4.3	***350.8***	0.90	Bajpai and Pradeep 2013 ^23^
pullulan:alginate (40:60)		98.0	***0.0***	***1.02***	Xiao and Tong 2013 ^22^
pullulan:alginate (60:40)		89.0	***0.0***	0.84	Xiao and Tong 2013 ^22^
pullulan		72.0	***0.0***	0.63	Xiao and Tong 2013 ^22^
gum arabic	10	4.9	***–14,233,103***	0.95	Tatar et al. 2014 ^24^
guar	20	3.2	***99.9***	0.99	Torres et al. 2012 ^25^
locust bean gum	20	4.1	41.0	0.99	Torres et al. 2012 ^25^
tragacanth	20	5.0	21.7	0.94	Torres et al. 2012 ^25^
xanthan	20	7.7	8.2	0.92	Torres et al. 2012 ^25^
apple pectin	25	4.4	1.5	0.97	Panchev et al. 2010 ^26^
citric pectin	25	4.0	1.8	0.99	Panchev et al. 2010 ^26^
sunflower pectin	25	4.4	1.9	0.94	Panchev et al. 2010 ^26^

a Values in bold italic font vary from the understood physical constraints of the model.

Baukh et al. assumed an instantaneous diffusion and uniform distribution of water, and hence they set the *C* value to 1. They approximated the maximum amount of water that can be held within the sorbing layer, *m*_max_, by substituting *a*_w_ = 1 and setting *C* = 1 in the GAB equation (eq 3), giving $m_{\max}=\frac{m_{\mathrm{mono}}\cdot K}{\left(1-K\right)}$ . The GAB isotherm (eq 3) was then normalized by *m*_max_ to obtain

$$\frac{m_{\mathrm{bulk}}}{m_{\max}}=\frac{a_{w}\left(1-K\right)}{1-K\cdot a_{w}}$$ 4

Equation 4 was used to obtain a value of *n*_max_ via the molar mass of water (18.02 g mol^–1^) to solve eq 2 numerically.

We extended the Baukh model by allowing water transport from one side of a multilayer structure to the other (removing the impervious surface). To stop the water-sorbing layer from drying out we added a second barrier layer in the structure, as is illustrated in Figure 2C, thereby making a trilayer system. The function of the first barrier layer is to prevent ingress of water from the high activity side, and the function of the second barrier is to prevent egress of water from the sandwiched sorbing layer. Configuring a barrier such that there is an intermediary water-sorbing middle layer alters the local water activity and Δ*a*_w_ in eq 1. When the sorbing layer has the same initial activity as does the water activity outside the barrier, the differential in water activity across the final barrier layer is zero, and thus the overall flux is zero. When water is sorbed into an intermediate layer, the local water activity will rise. The differential between the intermediary layer and the surroundings will begin to increase until eventually the system is in equilibrium, with the activity in the sorbing layer taking a value intermediate between the two sides (see Figure 2C).

The structure can be expanded from a trilayer into an *n*-layer, where *n* is an odd integer. The flux of water across the barriers can be represented by

$$J_{1}=\frac{p_{1}}{L_{1}}(a_{\mathrm{in}}-a\left(\chi \right)),,J_{2}=\frac{p_{2}}{L_{2}}\left(a\left(\chi \right)-a_{\mathrm{out}}\right)$$ 5

where the *J*_1_, *p*_1_, and *L*_1_are the flux, permeability (*D*·ρ), and thickness of the first barrier layer (the high activity side in Figure 2C). In a similar way, *J*_2_, *p*_2_, and *L*_2_ are the flux, permeability and thickness of the second barrier layer. Here, *a*_in_ and *a*_out_ are the water activities on either side of the multilayer system, and *a*(χ) is found from the inversion of the sorption isotherm. The absolute number of water molecules that pass through both barriers into the sorbing layer can be found from

$$\frac{\delta n}{\delta t}=A\left(J_{1}-J_{2}\right)$$ 6

where δ*n* is the change in the number of moles of water. By combining eqs 5 and 6, the change in water content can then be modeled by the difference in water activity between both sides of the sorbing layer at the water activity of the sorbing layer, as so:

$$\frac{\delta \chi }{\delta t}=\frac{a_{\mathrm{in}}}{\tau_{1}}+\frac{a_{\mathrm{out}}}{\tau_{2}}-a\left(\chi \right)\left(\frac{1}{\tau_{1}}+\frac{1}{\tau_{2}}\right)$$ 7

where there is a characteristic time for each barrier layer (τ_1_ and τ_2_). In our simulations we solved eq 7 numerically. Time points were chosen to be of sufficient length as to allow the assumption of instantaneous redistribution of water to hold, given the barrier thickness. The quantity of water transported was then calculated given the activity differential. The new mole fraction water content was converted to the new activity via the sorption isotherm normalized to the maximum amount of water that can be held in the system (*a*_w_ = 1)

$$m_{\max}=\frac{m_{\mathrm{mono}}\cdot C\cdot K}{\left(1-K\right)\left(1-K+C\cdot K\right)}$$ 8

As with the Baukh model, eq 3 is made dimensionless by eq 8 to give a mass fraction of water in the sorbing layer relative to the maximum (*m*_S_)­

$$m_{S}=\frac{m_{\mathrm{bulk}}}{m_{\max}}=\frac{\left(1-K\right)\left(1-K+C\cdot K\right)\cdot a_{w}}{\left(1-K\cdot a_{w}\right)\left(1-K\cdot a_{w}+C\cdot K\cdot a_{w}\right)}$$ 9

Equation 10 can be rearranged (in our case by aid of MATLAB) to calculate the water activity for use in eq 5.

By adding additional fluxes to eq 5, the number of layers in the multilayer design can be extended to an indefinite number of odd layers

$$J_{1}=\frac{p_{1}}{L_{1}}\left(a_{\mathrm{in}}-a\left(\chi_{1}\right)\right),J_{2}=\frac{p_{2}}{L_{2}}\left(a\left(\chi_{1}\right)-a\left(\chi_{2}\right)\right),\cdot \cdot \cdot ,\;J_{n+1}=\frac{p_{n+1}}{L_{n+1}}\left(a\left(\chi_{n}\right)-a_{\mathrm{out}}\right)$$ 10

where *n* is the number of sorbing layers in the system. The water content of each layer was calculated by eq 7 after substituting the water activities on either side of the sorbing layer. A five-layer structure is illustrated in Figure 2D. We made the same three assumptions in our model as Baukh et al.^14^ did. We also assumed that the GAB model can predict the maximum amount of water the material can hold. However, as *K* approaches 1, the GAB equation approaches the Braunauer-Emmett–Teller model (another common model for fitting isotherm data), and the sorption isotherm asymptotes at 1. The typical applicable range for the GAB model is usually from water activities from 0.05 to 0.90.^16^ As the *a*_w_ approaches 1, the model will likely overpredict the water-holding capacities. Alternatively, the maximum water-holding capacity can be experimentally obtained (by fully hydrating the sample) and used directly in the model.

## 3 Materials and Methods

In our experiments, we created three model systems, to represent various levels of ideality (see Figure 4). The most ideal system consisted of liquid glycerine sandwiched between acetate sheets. A second model system used two free-standing chitosan sheets sandwiched between three layers of acetate. The third model system is more practical for packaging applications. It was comprised of two waterborne barrier coatings interlayered with a cast film of chitosan

**Figure 4:**
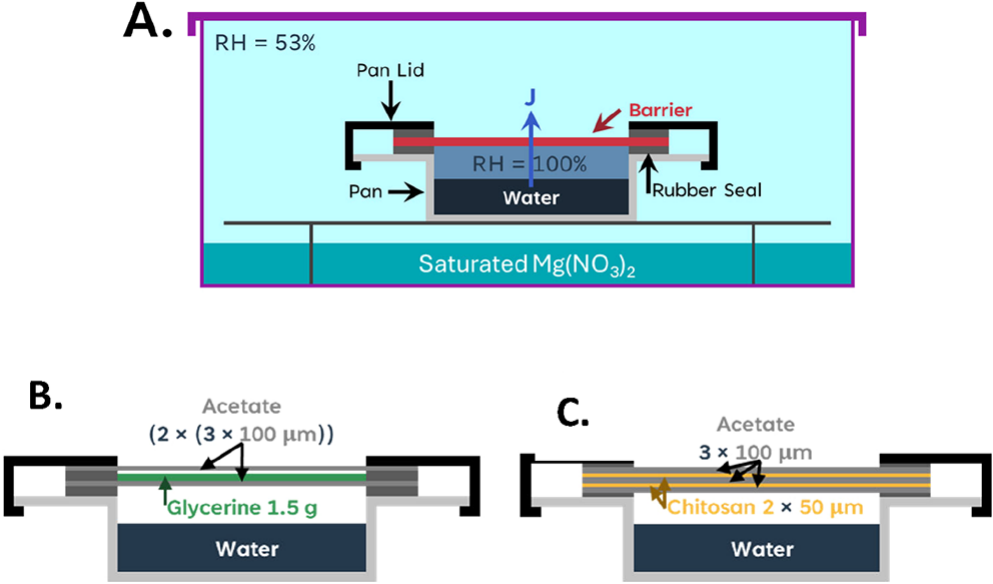
(A) Schematic of the conditions for monitoring the water vapor transmission rate of the Payne cups. (B) Schematic of the setup for the acetate and glycerine model system. (C) Schematic of the setup for the acetate and chitosan model system.

The water vapor transmission rates were determined by using Payne permeability cups with an open area of 10 cm^2^ (TQC-Sheen Industrial Physics, Thame, UK). To improve the reproducibility of the Payne cups, we added 1.5 mm rubber seals (RA components, Corby, England, UK) cut to flat O-rings by use of a cutting machine (Cricut, South Jordan, USA). Silicone vacuum grease (RA components, Corby, England, UK) was used on the O-rings to improve the seal. A constant water activity differential was maintained by filling the cups with deionized water and storing them in a bespoke humidity box regulated to a relative humidity (RH) of 53% by use of saturated aqueous solution of magnesium nitrate (Mg­(NO_3_)_2_) at an ambient temperature of 20 ± 2 °C. A schematic of the setup is given in Figure 4A. The mass of each filled cup was recorded at regular intervals, typically daily, on a digital balance to the nearest 0.1 mg.

For the first model system, 100 μm thick cellulose acetate sheets (Hobbycraft, Dorset, UK) as barrier layers. We used acetate sheets in our model system because their permeability is not too low (which would make the experiments excessively long) and they can be obtained with consistent properties and dimensions, making them suitable for permeability measurements. The sheets were cut to an appropriate circle to fit a 10 cm^2^ Payne permeability cup using a cutting machine (Cricut, South Jordan, USA). The acetate sheets were equilibrated for several days at 53% RH. The multilayer was made using three sheets of 100 μm cellulose acetate sheets stacked on top of each other, which was sandwiched between rubber O-rings. Then 1.5 g of predried (105 °C, >16 h) glycerine was spread on the acetate in the reservoir inside of the O-ring. Next, an additional set of three sheets of 100 μm acetate were place above the glycerine, before sealing all layers in the Payne cup. This structure is presented in the schematic in Figure 4B.

For the second model system, low molecular weight chitosan powder (10 cP Crystal, CuanTec Ltd., Argyll, Scotland, UK) was dissolved in 2.5% (w/v) acetic acid solution to give a 2.5% (w/v) chitosan solution. Freestanding chitosan films were made by using a doctor blade set to 1 mm with a draw-down at a low controlled speed of 5.5 mm s^–1^ on a coating apparatus (Elcometer Ltd., Manchester, UK). The chitosan solutions were cast onto clean glass plates, and then dried in a convection oven (Sanyo, Tokyo, Japan) for 30 min at 90 °C. The film was left to cool and then peeled from the glass substrate. Chitosan films were cut to size manually with scissors to avoid tearing. A five-layer barrier system was made by alternating 100 μm thick acetate sheets and free-standing chitosan films (50 μm thick) as is indicated in the schematic in Figure 4C.

For the third model system, the barrier coatings were deposited from a waterborne styrene-acrylic copolymer latex, synthesized by emulsion polymerization, and formulated with thickeners, wetting agents and hydrophobic additives, as provided by SciTech Adhesives Systems Ltd. (Flint, Wales, UK). The solids content of the formulation was 32 wt %. The glass transition of the primary copolymer was approximately at 0 °C. Office paper (120 gsm copy paper, Rayman, Crewe, UK) was used as a substrate. The dispersion was coated on the paper using a 60 μm Meyer bar and film-formed in an oven at 90 °C for 10 min. The resulting coating was hydrophobic, with a water contact angle of 99°. Samples were cut to an appropriate circle to fit a 10 cm^2^ Payne permeability cup using a cutting machine (Cricut, South Jordan, USA). The multilayer system was made by placing the barrier-coated paper on top of a rubber seal (paper in contact with the rubber), and then pressing a free-standing chitosan sheet onto the barrier coating. Next, a second barrier-coated paper placed on top of the chitosan with the barrier coating in contact with the chitosan.

Sorption isotherms of chitosan (powders and free-standing films) were obtained by measuring the mass uptake with increasing relative humidity (RH) using a microbalance (DVS, Surface Measurement Systems, London UK). Humidity boxes containing saturated salt solutions were also used to set the RH to allow gravimetric measurements on bulk samples. Water contents of chitosan powders were measured via thermogravimetric analysis (TGA 550, TA Instruments, New Castle, DE, USA) by heating the sample from ambient to 150 °C in a N_2_ atmosphere. The mass loss after a 30 min isothermal hold at 150 °C was used. The mass sorbed at a given RH (whether from the DVS or the humidity boxes) was fitted with the GAB eq (eq 3) using MATLAB software. The ‘fit’ function was used to apply a nonlinear least-squares method to obtain a fit to the data for the values of *C, K* and *m*_mono_. The fits were constrained within the physical limits of 0 < *C* < 100, 0 < *K* < 1, and 0 < *m*_mono_ < 500 using the ‘trust-region-reflective’ algorithm.

## 4 Results and Discussion

### 4.1 Simulations from the Model

Simulations of various multilayer barrier systems (called multilayers hereafter as shorthand) were conducted to evaluate the effect of the various variables in the model. These variables include the thickness (*L*_B_) and permeability (*p*) of the barrier, the maximum mole fraction water-holding capacity of the sorbing layer (χ_max_), the number of layers, the ultimate activity differential on either side of the layers, as well as the initial water activity of the sorbing layers. In practice, χ_max_ is not readily known but needs to be calculated from the sorbing layer thickness (*L*_S_), its mass density, ρ_S_, and the maximum mass ratio of water, *m*_max_, obtained from the GAB values governing the sorption isotherm (eq 8) for the sorbing material, as follows:

$$\frac{\chi_{\max}}{A}=\frac{m_{\max}.\rho_{S}.L_{S}}{M}$$ 11

where *M* is the molar mass of water (18 g mol^–1^). Unless stated otherwise, model simulations were executed with the conditions as listed in Table 2. The values chosen for *m*_mono_, *C*, and *K* are typical for a hydrophilic biopolymer, such as chitosan. Each variable was systematically varied to determine its effect on the water flux of the total system in an attempt to better understand how to design an ideal multilayer barrier system.

**Table 2:** Default Parameters Used in Model Simulations

**parameters**	**value**
total layer number	5
individual barrier layer thickness (*L*_B_)	100 μm
individual barrier permeability (*p*)	1 nmol m^–1^ s^–1^
individual sorbing layer thickness (*L*_S_)	100 μm
individual sorbing layer density (ρ_S_)	1 g cm^–3^
sorbing layer *m*_mono_	10 g g^–1^
sorbing layer *C*	10
sorbing layer *K*	0.85
internal water activity (*a*_in_)	1
external water activity (*a*_out_)	0.53
preconditioned sorbing layer activity (*a*_s_)	0.53

The typical output of the model is given as the mass loss per area as illustrated in Figure 5A. The gold dashed line is the mass loss of the equivalent barrier system when no sorbing layer is present (i.e., 3 × 100 or 300 μm of a barrier with *p* = 1 nmol m^–1^ s^–1^). The solid blue line is the prediction when the sorbing layer is included. The gradient of the curves provides the total water flux through the barriers, as would be measured in a gravimetric experiment. As can be seen in the figure, there is a delay in water loss through the barrier (with a low water flux) until eventually there is parity in water flux between the two curves; that is, the system reaches an equilibrium and there is no longer an effect from the sorbing layer. Astute readers will notice that time to parity can be calculated by determining the difference between the system comprised of the equivalent thickness of continuous barrier i.e. no sorbing layers (hereafter referred to as equivalent barrier) and the multilayer system once equilibrated. A simple series of logical tests were performed (in MATLAB) to calculate this delay time (*t*_d_) (see the description in the Supporting Information and Figure S1). We define equilibrium as being reached when the water mass fraction (*m*_S_) is greater than 99% of the final amount (*m*_end_) as is demonstrated in Figure 5B. A linear fit was then applied to the multilayer data, as indicated in Figure 5A, and the *x*-axis intercept was taken to be the delay time.

**Figure 5:**
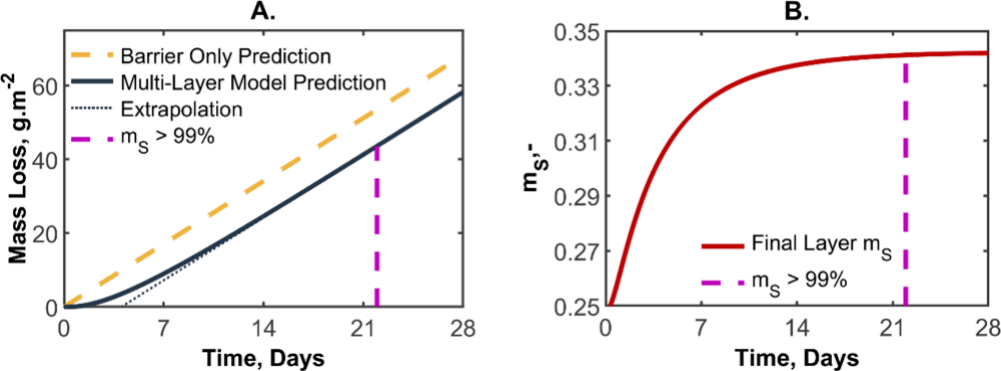
(A) Mass loss of a five-layer system (solid blue line) and an equivalent barrier system without the sorbing layers (dashed gold line) as predicted by the proposed model. The dashed purple line indicates the time when an effective equilibrium (99%) has been reached. The dotted blue line shows the linear fit to the multilayer prediction when at an effective equilibrium, which is used to find the delay time. (B) Mass fraction water content normalized by the fully hydrated layer (*m*_S_) of the final layer in a multilayered barrier system. Effective equilibrium is said to be reached when the *m*_S_ is 99% of the difference between the initial and end values, at the time indicated by the dashed purple line.

An interesting observation can be made. No matter which sorbing layer is considered, parity with the hydrophobic barrier will always be achieved. This indicates that incorporating sorbing layers into the design in the short term can improve a traditional single layered system which is described by its thickness and permeability. In the indicated example, packaging made with a multilayer barrier system is approximately doubly as effective as an equivalent barrier at day 7. For short shelf life products (such as milk), such a delay is of high utility. Hereafter, we use the delay time as a measure of the effectiveness of the barrier systems. For specific products with a short shelf life, one might instead look at the ratio between the mass loss from an equivalent barrier and the multilayer on a given day, e.g., 50% less mass loss on day 7.

Each of the parameters in Table 2 were tested, either individually or in combination with other variables, to fully evaluate which conditions are optimal for an improved barrier system design. The outcomes are presented in the following sections.

#### 4.1.1 Effects of Physical Properties on the Water Flux

Initial simulations investigated the effects of the barrier thickness and its permeability. The primary aim was to verify that the simulations functioned correctly. These results are presented in the Supporting Information as Figure S2. As is expected from eq 1, increasing the equivalent barrier thickness or decreasing its permeability were found to decrease the water mass loss per unit area.

The standard multilayer design, given in Table 2, was altered to change the thickness of each layer (three barrier layers or two sorbing layers) to various total thicknesses, as is indicated in Figure 6A. For the blue curve, the sorbing layer thickness was fixed (*L*_S_ = 100 μm, total thickness 200 μm) and the cumulative barrier thickness was varied from 1 μm to 1 mm (*L*_B_ = 0.33–333 μm). A linear fit through the origin was obtained in MATLAB and is presented in Figure 6A as a dotted blue line, indicating a straight-line dependence of the delay time on the barrier thickness. This indicates that a thicker barrier layer makes the same sorbing layers more effective. An inverse dependence of the delay time on the barrier permeability was found (see Figure 6B). By considering both the barrier layer thickness and its permeability, the addition of a sorbing layer can improve an already weakly permeable barrier system by decreasing the mass loss rate for a certain period of time.

**Figure 6:**
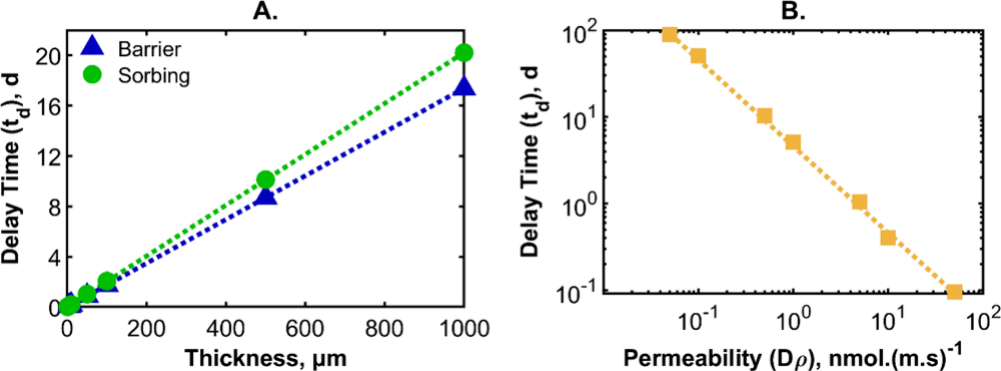
(A) Delay time as a function of the cumulative thickness of the barrier layers (blue triangles) and sorbing layers (green circles) in a five-layer structure (as described in Table 2). The dotted lines are linear fits passing through the origin. (B) Delay time as a function of the barrier layer permeability with the standard parameters in a five-layer structure. The dotted line shows an inversely proportional fit.

Figure 6A shows the effect of increasing the total sorbing layer thickness (0.5 ≤ *L*_S_ ≤ 500 μm) while holding the barrier layer thickness constant (*L*_B_ = 100 μm). The dependence of the delay time on the sorbing layer thickness is linear, as shown by the green line. This is a useful finding. Water flux through barrier layers with a relatively low thickness can be decreased by adding thicker sorbing layers. In the case of water-sorbing biodegradable biopolymers, thicker sorbing layers will not pose the same environmental impact as will thicker barrier layers of petrochemical origin. Figure 6A,B presents the time delays, however the absolute mass loss across the barrier is also important.

The mass losses at the delay times for multilayers with varying barrier and sorbing layer thicknesses are given in Figure 7A and for varying permeabilities in Figure 7B. The mass losses on day 7 for the simulations of varying barrier and sorbing layer thicknesses is given in Figure 7C. The data in the three figures show that the mass of water lost is set by the sorbing layer thickness and the delay time is set by the thickness and permeability of the barrier layer. For the varying barrier thickness and the varying permeability, the amount of water lost between *t*_0_ and *t*_d_ is fixed. Assuming the barrier layer is not altered, the delay time can be used to compare any amendments to the sorbing layer without needing to consider if the mass loss is also changing. Conversely, the changes to sorbing layer do alter the mass loss; better sorbing layers give longer delay times. However, more water also passes through the barrier compared to a variable barrier layer. Improvements to the barrier system can be designed in terms of aiming to have a delay of a certain number of days or for an improvement over a certain time period.

**Figure 7:**
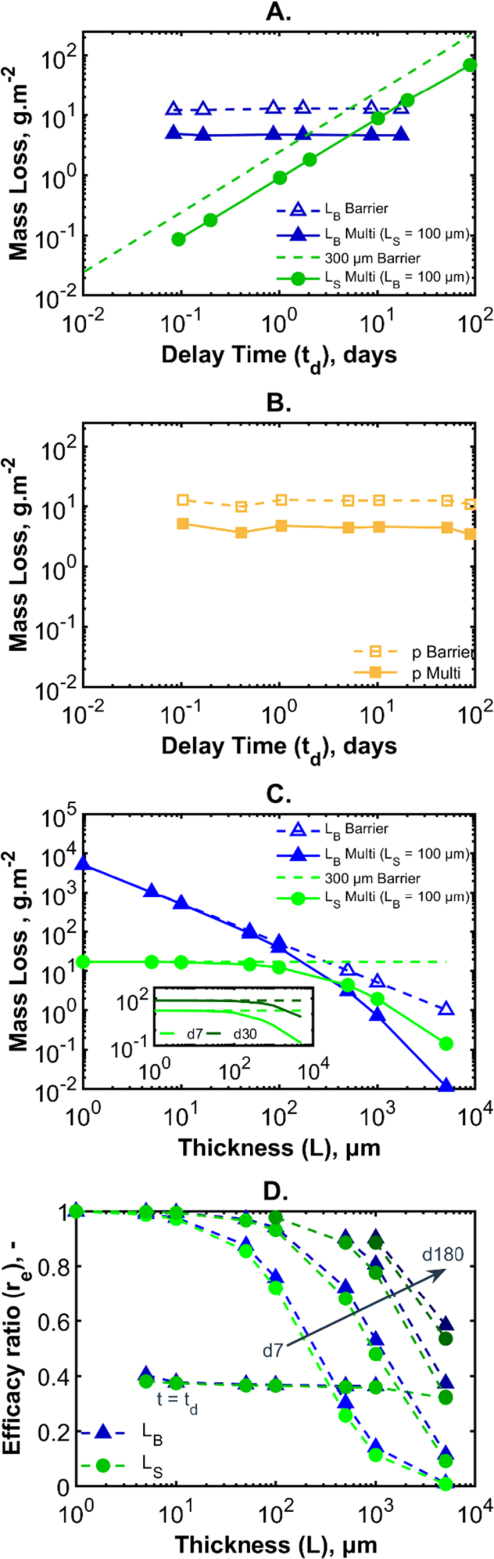
(A) Water mass loss as a function of the delay time for various thickness systems. Dashed lines with open symbols indicate the mass losses from the equivalent barrier layers (=barrier). Solid lines with filled symbols indicate the mass losses of multilayer systems including the sorbing layers (=multi). Variable thicknesses of barrier layers (*L*_B_) are presented in blue, and variable thicknesses of sorbing layers (*L*_S_) are presented in green. (B) Water mass loss as a function of delay time for simulations of various barrier permeabilities. Dashed line with open symbols indicates the barrier layer equivalent mass losses. Solid line with filled symbols indicates the mass loss of multilayer systems (including a sorbing layer). (C) Water mass loss in 7 days as a function of the layer thickness. Blue triangles represent the variable barrier layer thickness; green circles represent the variable sorbing layer thickness. Open symbols (with dashed lines) represent the mass loss for the equivalent barrier (no sorbing layer). Filled symbols (with solid lines) represent the multilayers. The inset shows a comparison of the mass loss of different thicknesses of the sorbing layer on day 7 (d7) and day 30 (d30). (D) Efficacy ratio (the ratio between the mass loss of the equivalent barrier and the multilayered system) as a function of sorbing layer thickness (shades of green) and barrier layer thickness (shades of blue) at different time points. Various times are considered from day 7, day 30, day 90, and day 180. The efficacy ratio (*r*_e_) at the delay time is written on the curve *t* = *t*_d_.

Figure 7C compares the mass loss of the multilayer design on day 7 (d7) to the barrier equivalent. The green data show the effect of variable sorbing layer thickness and the blue curves of varying barrier layer thickness. The inset compares d7 and day 30 (d30). The pattern is the same for the varying barrier thickness with the day 30 data having a higher mass loss at that thickness. The difference between the equivalent barrier and the multilayer is also lower at the same thickness. As to be expected for the equivalent barrier, increasing the *L*_B_ decreases the mass loss. When sorbing layers are included and the *L*_B_ is fixed at 100 μm, the mass loss is independent of the sorbing layer thickness. When incorporating the effect of the sorbing layer, there appears to be a critical thickness (tens of μm for the barrier layers used here) before which any improvements are seen, i.e. the multilayer offers no improvement on the equivalent barrier. Changing the day of interest transposes the curves without altering their overall shape. This indicates that the expected improvement to the barrier system diminishes the longer that the simulation is run.

The ratio between the mass loss of equivalent barrier compared to a multilayered system indicates the effectiveness of the multilayer system at a specific time. We term this the barrier efficacy ratio (*r*_e_), where the lower is the value, the greater is the efficacy of the multilayer system.Figure 7D gives *r*_e_ at *t*_d_, as well as at *t* = 7, 30, 90, and 180 days. The efficacy ratio at the delay time (*t*_d_) was found to range from 0.3 to 0.4 with a weak negative dependence on the layer thicknesses (*L*_B_ and *L*_S_). Likewise, there was a weak positive dependence of the efficacy ratio on the barrier permeability (not shown). When changing the day of analysis, the *r*_*e*_ is transposed on the *x*-axis. At each day, there is an initial plateau where increasing *L*_B_ and *L*_S_ do not add any benefit to the barrier. As the thicknesses are increased, the efficacy ratio approaches a value of zero where, the multilayer effectively does not allow any water to pass through. Typically, this is when the barrier is very thick, on the order of a few mm. The relationship between *r*_e_ and the layer thickness is sigmoidal, indicating that after a certain thickness there are diminishing returns in terms of mass loss savings. The blue curves are all comparable, as the mass loss of the equivalent barrier on any given day is set by the total barrier layer thickness. For variable *L*_*B*_ the absolute amount of mass loss is reduced as the barrier thickness increases. Therefore, the system as a whole is less permeable, and the sorbing layer further decreases the mass loss.

We hypothesized that the distribution of thickness within the barrier will also impact the water flux through the system. To investigate the distribution of thicknesses, a five-layer barrier design was chosen consisting of three barrier layers with a total thickness of 600 μm and two sorbing layers with a total thickness of 400 μm. The isotherm and barrier properties and the overall activity differentials were fixed as per Table 2. The thicknesses were incremented in steps of 100 μm to give 30 different permutations. They were grouped into three according to the distribution of the sorbing layer. A schematic is given in Figure S3 for a visual representation of the permutations. If, as we hypothesize, the distribution affects the barrier properties of the multilayer system, the delay time will be different per each permutation.

The delay times obtained from investigating the layer thickness distributions are presented in Figure 8A with the highest and lowest delay times presented in Figure 8B.

**Figure 8:**
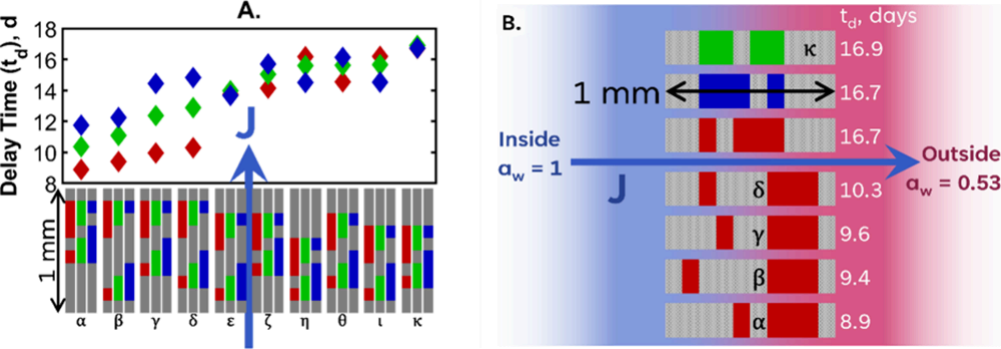
(A) Delay times obtained for the various thickness permutations. The thickness distribution of barrier layers (in gray) is identified by Greek letters α through κ in the schematic (under the graph). The red, green, and blue diamonds refer to the distribution of the sorbing layer thicknesses as illustrated in the schematic. The water flux direction is indicated by a blue arrow. (B) Schematic diagram of the permutations leading to the lowest and highest delay times. The schematic lettering matches what is in panel A, and the RGB colors match the sorbing layer thicknesses that are illustrated in panel A.

Some apparent trends can be seen in Figure 8A:

- The worst four performing multilayers (in the red group) each have
  - a final barrier (layer five) that is thin (100 μm).
  - a second sorption layer (layer four) that is thick (300 μm).
- The effect of the distribution of the sorbing layer varies greatly with the distribution of the thickness of the barrier layers, with groups ε and κ having a negligible influence on the multilayer performance and others (such as groups γ and δ) increasing the effectiveness by 50%.
- In most instances, having the thicker sorbing layer close to the high-activity side improves the multilayer performance. This trend is reversed when the first barrier layer (layer one) is only 100 μm thick, as is the case in groups η and ι.
- The most effective barrier permutation is in group κ which has this structure:
  - a 200 μm thickness of the barrier along the high-activity interface (layer one);
  - a middle barrier layer with 100 μm thickness (layer three);
  - a 300 μm thickness barrier along the low-activity (drier) interface (layer five).

The effect of the sorption layer thickness distribution is negligible in group κ. As the middle barrier layer is relatively thin, the diffusion between the two sorption layers is rapid, and the system moves closer to a trilayer design. These results are discussed in more detail in Section 4.1.3, which evaluates the effect of layer number.

The least well performing permutations all have a thin final barrier on the dry side (groups α-δ). The low performance can be explained by there being a minimal resistance between the final sorbing layer (layer four) and the ambient humidity. In these configurations the activity differential is the highest. Any water that is in layer four will quickly diffuse through the final barrier. This concept also explains why the distributions of thickness between layers two and four (the two sorbing layers) play such a role in the delay time. When layer two is thin, a small amount of water can be sorbed there before reaching equilibrium with layer four. When the first barrier (layer 1) is thin, we assume that the first sorbing layer (2) equilibrates rapidly with the inside of the container and the system effectively moves closer to a trilayer system. Thus, when the sorbing layer thickness distribution is skewed to be thinner near the inside (layer 2), the second sorbing layer (layer 4) is thicker and able to hold more water. This is likely to be the explanation for why the red sorption layer design in groups η and ι outperforms the blue (contrary to all the other permutations).

From these simulations we deduce that the most effective distribution of thickness is to have a thicker barrier on both outer sides of the multilayer and an equal distribution of thickness of the sorbing layers. As with the previous analysis of variable thicknesses, *r*_*e*_ was found to lie between 0.33 and 0.4.

#### 4.1.2 Changes to the Water Content of the Sorbing Layer

Section 4.1.1 investigated the effects of thickness of the layers and the permeability of the barrier layers. Next, the differential in the chemical potential of water (activity) was varied to see the effect. Two separate trials were run. In the first trial, the *m*_mono_, *C* and *K* values of the sorbing layers were adjusted to change the activity differentials. The second trial altered the initial activity of the sorbing layers (their precondition) as a means to modulate the activity differential. In both cases, the barrier layers were kept constant, so the changes in delay time are comparable between the same trials.

For the first trial, a five-layer barrier system was used as per Table 2, with the exception that the sorbing layer thicknesses were increased to 200 μm to accentuate the effect of amending the GAB values. The *m*_mono_ value was set at 1, 10, 15, or 50 g/g (%); the *C* value was set at 2, 5, 15, 50 or 99; and the *K* value was set at 0.5, 0.625, 0.725, 0.825, or 0.95. Equation 4 is not defined at *C* = 1 or at *K* = 1. The ranges proposed are sensible when considering the physical constraints of the GAB equation. The delay times obtained from these simulations are presented in Figure 9A, and the mass losses on day 7 are in Figure 9B. Consistent with the effects on the sorption isotherm, the value of *m*_mono_ has the greatest effect on the delay time, followed by the *K* value. The *C* value appears to have little role to play in determining the delay time (Figure 9A). The simulation shows that significant improvements can be made to a multilayer system by including a highly sorbing material (such as glycerine, as indicated in the figure) into a multilayer design. The dependence of *t*_d_ on *m*_mono_ and *K* indicates that the overall water-holding capacity is the key in determining the water transport. In Figure 3, it is seen that the *C* value alters the initial slope of the sorption isotherm. One might have expected that this might affect the water flux well before there is parity between the mass loss of the barrier equivalent and the multilayer design. In such instances, say on day 7, one might expect the effect of *C* to be more pronounced in the simulations with high *m*_mono_ and *K* values. This was not observed in Figure 9B; thus, we conclude that the water sorption at high activities dominates; changes to the sorption isotherm shape at low humidities do not significantly impact the water mass loss. The efficacy ratio, *r*_e_ was found to fall between 0.25 and 0.41 across the range of GAB parameters. A surface plot of the data is presented in Figure S4 in the Supporting Information. There is a weak inverse relationship between *r*_*e*_ and *m*_mono_ values.

**Figure 9:**
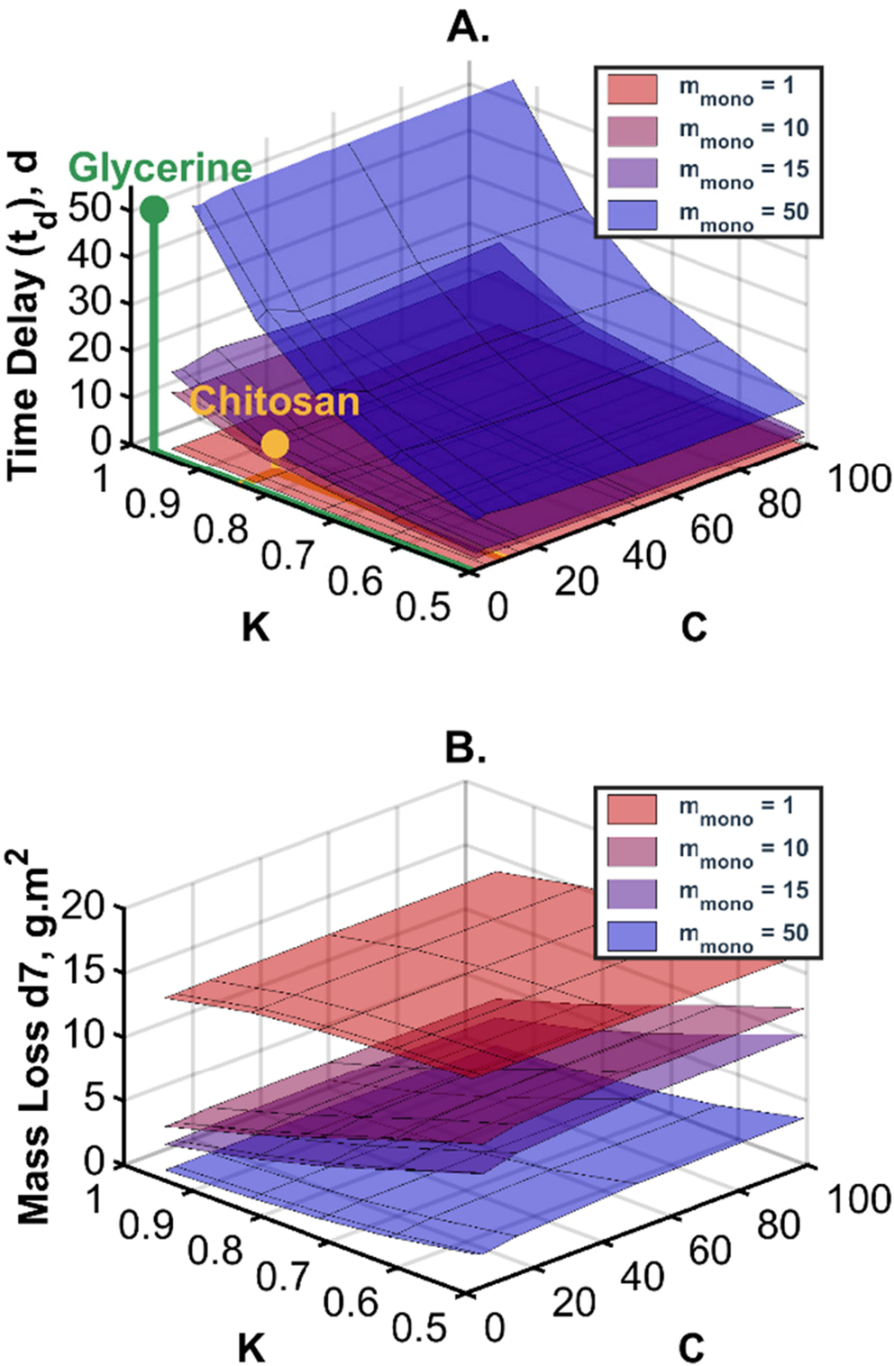
(A) Surface plot of the time delay of varying *m*_mono_, *C*, and *K* values used in the GAB equation. The predictions using the isotherm data of glycerine (green) and chitosan (gold) are indicated as points on the curve. (B) Surface plot of the water mass loss on day 7 obtained when varying the *m*_mono_, *C*, and *K* values used in the GAB equation. The lowest mass loss is achieved with the highest value of *m*_mono_.

The sorption layer thickness, its density, and the sorption isotherm define the overall water-holding capacity before saturation. Separately, we changed the preconditioned amount of water that was initially in the sorbing material. If we consider the sorbing layer to be a tank, then *L*_S_, ρ_S_, and the GAB parameters affect the volume of the tank; the preconditioned *a*_w_ then sets the initial fill level prior to the simulation running. The initial *a*_w_ will influence the local flux between layers. We hypothesize that although the main driver for the water flux is the Δ*a*_w_ on either side of the multilayer barrier as a whole, the local differential from one side of a barrier layer to another is also important. Then the total flux can be modulated depending on which layers are hydrated in the multilayer. The null hypothesis is that the average initial activity (of all the individual sorbing layers) of the multilayer system is the key driver; then the positioning of the prehydrated layers does not affect the overall flux. To test this hypothesis, we ran simulations of a nine-layer system as per Table 2, although *a*_s_ of each of the four sorbing layers was set to either 0, 0.5, or 1. A total of 64 simulations is represented schematically in Figure 10A, considering the permutations of *a*_s_. Figure 10B shows examples of combinations providing different average values of *a*_s_ ranging from fully dry (0) to saturated (1). As the barrier layers were not altered, the delay time is a fair representation of the effect of the sorbing layer preconditioning, and the results of the simulations can be readily compared.

**Figure 10:**
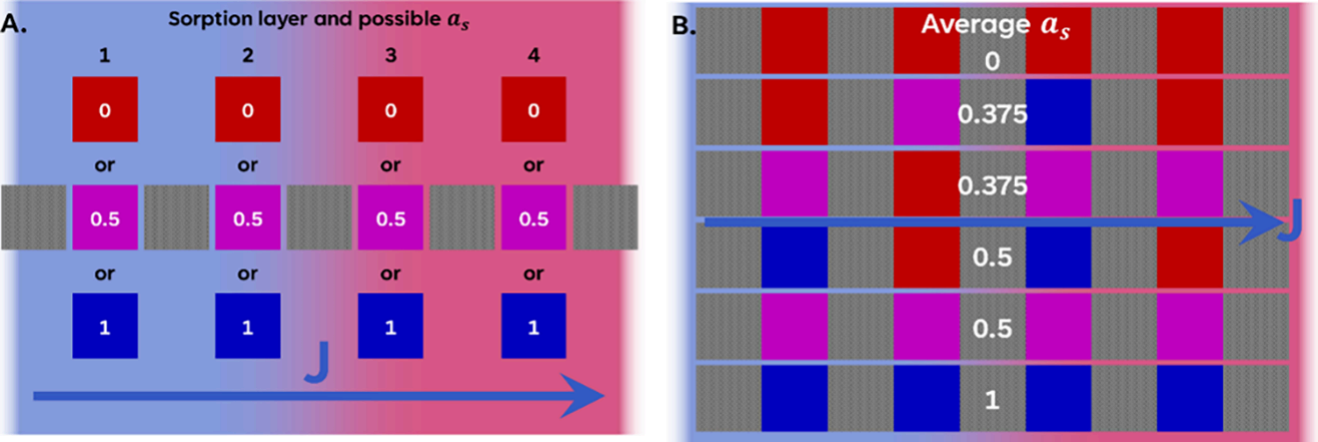
(A) Schematic diagram of the different preconditionings of the sorbing layers in a nine-layer structure having *a*_s_ values of either 0, 0.5, or 1. The gray squares represent the barrier layers. (B) Examples of different average water activities ranging from 0 to 1, which are obtained by the selection of preconditioned activity in the individual sorbing layers, as illustrated.

To test our hypothesis, we condensed all the simulations to a master plot of delay time as a function of average water activity in a nine-layer structure, as shown in Figure 11A. For a given average water activity, there are multiple combinations of preconditioned sorbing layers that can be used. The range of the delay times (i.e., the maximum and minimum values) is indicated with error bars on each data point. The number of combinations for each activity range is as high as 19 for an average activity of 0.5, but there is only one combination for average activities of 0 and 1. If the null hypothesis is true, all combinations would yield the same delay time. This is not the case. We thus deduce that the positioning of the preconditioned sorbing layers is of greater significance than the average *a*_s_. Indeed, the effect of position is such that the highest delay time for an average *a*_s_ of 0.75 (nearly saturated) is greater than the lowest delay time found for an average *a*_s_ of 0.25 (dried)–albeit by just 0.7 days.

**Figure 11:**
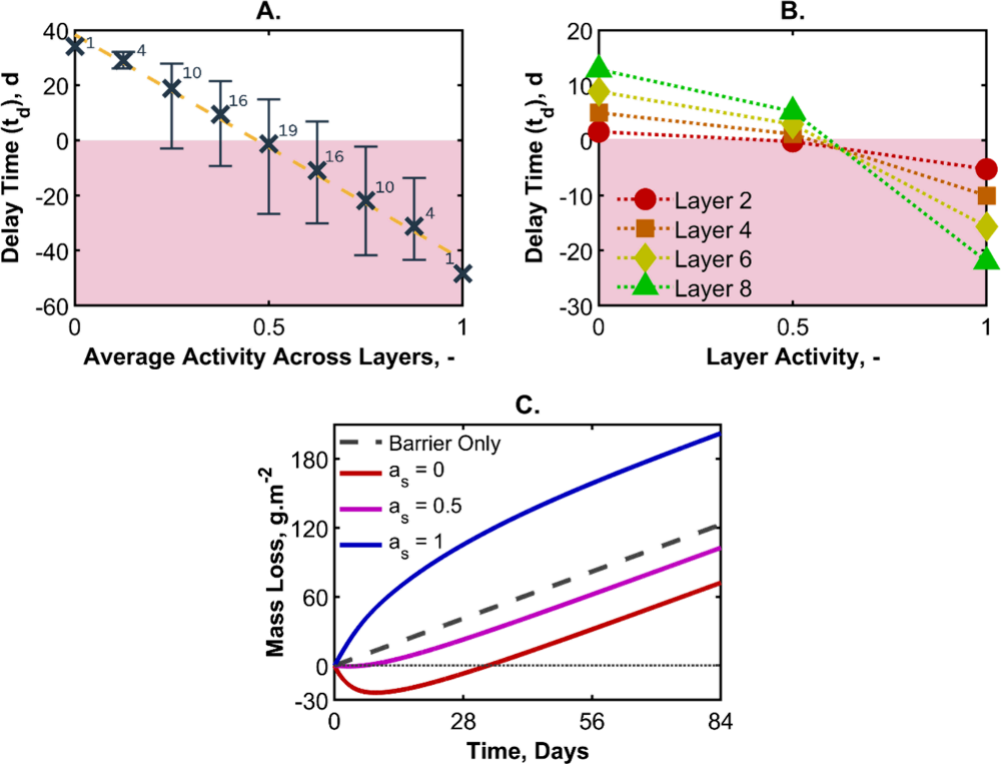
(A) Delay times of the average preconditioned water activity of four sorbing layers in a nine-layer system. Error bars indicate the absolute deviation from the mean. The numbers of activity combinations are presented next to each data point. The red-shaded area shows when the multilayer performs worse than the equivalent barrier system. (B) Delay times of each preconditioned layer independent of the conditioning of the other layers when set to water activities of either 0, 0.5, or 1. The red-shaded area indicates when the multilayer performs worse than the equivalent barrier system. (C) Mass loss as a function of time for a nine-layer system, where each of the four sorbing layers are preconditioned to a water activity, *a*_s_, of either 0, 0.5, or 1. When *a*_s_ = 1, there is a negative delay time (worse than the equivalent barriers).

To further understand how the *a*_s_ affects the *t*_d_, the data were condensed to show the delay time of each individual sorbing layer for three different precondition activities, independent of the activity of other layers. The results are presented in Figure 11B. It is evident that the preconditioning of the final layer is main determiner of the overall effectiveness of the barrier system. These results resonate with the thickness permutation experiments presented in Figure 8 where the final sorbing layer often has the greatest influence on the delay time.

In the previously presented simulations, the curves of mass loss against time followed the same shape, and the delay time was the main variable. In these simulations, the overall shape of the curves is now altered. Previously, the sorbing layers had been preconditioned to the same activity as *a*_out_, and the sorbing layer’s water sorption was confined to *a*_out_ ≤ *a*(χ) ≤ *a*_in_. In these present simulations, this constraint no longer applies, and the layer activity can be higher or lower than either side of the multilayer. In some circumstances, more water escapes through the final barrier when compared to the equivalent barrier without sorbing layers, particularly when the final sorbing layer is initially fully hydrated. These principles are illustrated in Figure 11C, where the initial activity across the four sorbing layers was set to *a*_s_ = 0, *a*_s_ = 0.5 or *a*_s_ = 1. The blue line (where each layer is set to an *a*_s_ of 1) loses more water than the equivalent barrier. Delay times less than 0 are shaded red in Figure 11A,B.

In contrast to the fully hydrated sorbing layers, sorbing layers in the multilayer could be fully dehydrated to such a degree that they sorb water from both sides of the overall multilayer (Side 1 and 2 in the schematic Figure 2D) and therefore the system gains water rather than losing it. Water mass gain is demonstrated by the red curve in Figure 11C which represents fully desiccated sorbing layers in a nine-layer stack. In principle, the maximum mass gain prior to mass loss is a function of the water-holding capacity (*L*_S_, ρ_S_, and the GAB parameters) and the difference between *a*_s_ and *a*_out_. This effect is, however, only achieved if all the sorbing layers are equalized to the same *a*_s_.

As with the delay time, the maximum mass gain is not solely a function of *a*_s_ and *a*_out_. When averaged, the data do not collapse. See Figure 12A. As with the delay time, the mass gain appears to be highly dependent on which layer is preconditioned to a low activity. The final layer has the greatest effect on the maximum mass gain of the multilayer, as indicated in Figure 12B. Where the layer preconditioning is variable, but the average layer activity is constant, the highest number of dry layers (with a preference for the eighth layer being dry) has the greatest mass gain. While the delay time is calculated from when the system is in equilibrium, the maximum mass gain is not. The shape of the curves obtained from these simulations can be quite unique. Some examples are presented in Figure S5 in the Supporting Information. Indeed, some preconditioned multilayers (low activity) start with a mass gain but eventually fare worse than the equivalent barrier. With the exception of these examples, the ratio between the delay time and the time of maximum mass gain was found to be between 0.2 and 0.4. No pattern or order was found when investigating the maximum mass gain relative to the mass loss of the barrier equivalent nor the mass loss of the multilayer on the delay time.

**Figure 12:**
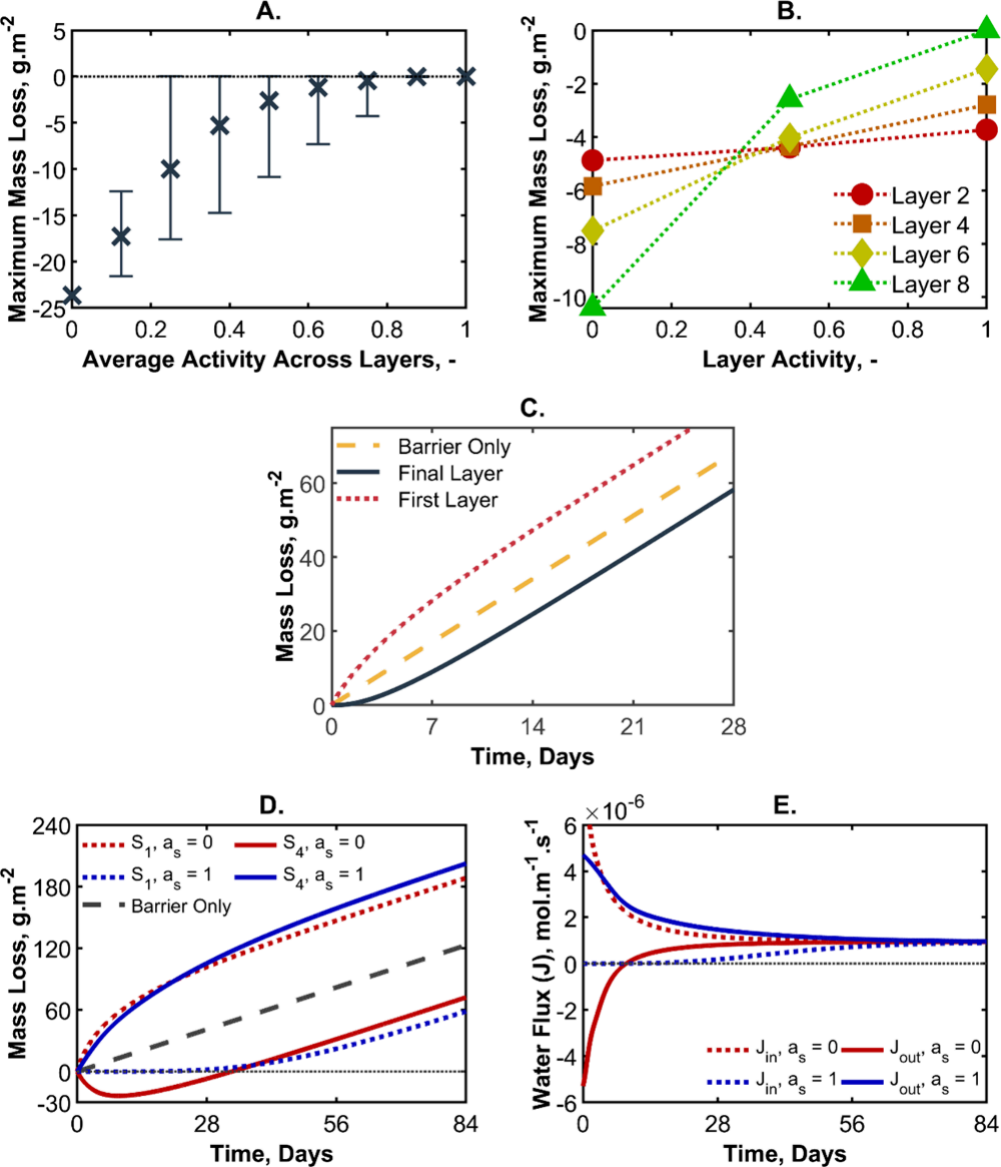
(A) Average minimum mass loss (mass gain) from the system for the average sorption water activity of the system by preconditioning the sorbing layers to less than the water activity of the low-activity side (side 2 in Figure 2D). (A negative mass change means that the multilayer is absorbing water from the low-activity side (humid air).) Error bars indicate the absolute range of deviation from the mean; *y* = 0 is indicated by a dotted line for reference. (B) Average maximum mass loss as a function of the sorbing layers’ preconditioned water activity: *a*_s_ of 0, 0.5, or 1. (C) Water mass loss from the default model prediction with the parameters found in Table 2 expressed for both the first and the final (fourth) sorbing layers in comparison to the equivalent barrier. (D) Mass loss of the first and final sorbing layers over time for two preconditioning levels. The first sorbing layer, *S*_1_, is indicated with a dotted line and the fourth and final sorbing layer, *S*_4_, is indicated with a solid line for a multilayer where the sorbing layers are preconditioned to either an *a*_s_ of 0 (red) or 1 (blue). (E) As for panel D but showing the water flux into the first sorbing layer (*J*_in_) or out of the final barrier layer (*J*_out_) over time.

Until this point, the effectiveness of the barriers has been considered in the context of the water evaporating out of the final layer, which is typically how a barrier system is evaluated practically. A container is weighed at regular time intervals, and the mass is recorded. In our analytical model, however, we can analyze the flux of water across specific individual barrier layers. Now we propose an alternative way of interpreting our results. We analyze the flux of water from inside the container (on the high activity side) into the first sorbing layer (*J*_in_), in comparison to the flux out of the final barrier layer into the environs with low activity (*J*_out_). In this way, we can monitor the change of a liquid product inside the container. When the aim is to limit the mass loss of an aqueous liquid from inside the container itself (and not through the packaging), then the system design must be reconsidered. In these instances, a fully hydrated barrier coating will limit the mass loss from the product.

Figure 12C shows an example of a five-layer multilayer with the specifications found in Table 2, in which the flux of water going into the first sorbing layer is far higher than the flux going out of the final layer. Although water is not being lost to the exterior during the first few days, it is being lost from the interior. Recall that the noticeable delay time refers to water loss to the surroundings. As can be seen from the example in Figure 12C, the findings discussed in the beginning of this subsection (presented in Figure 11) need to be re-evaluated to lower the transport from the interior.

For the discussion in section 4.1.1 the delay time signifies a reduction in mass loss of the product from the interior through the packaging. The results in this section (Figure 11), however, particularly with the very dry sorbing layers, might lead to the product losing mass from the interior more quickly than if their layers were hydrated. Figure 12D,E shows the change in water mass and the flux, respectively, for a nine-layer system when all sorbing layers are either fully dried or fully hydrated. Even though the fully hydrated layers (*a*_s_ = 1) allow the loss of more water through the final barrier, water is not lost from side 1 (the interior) for ∼28 days. Until that point, the water is only lost from the multilayer packaging. As previously stated, packaging performance is evaluated by overall mass loss of the barrier with its content and therefore testing flux through the individual layers nondestructively is nontrivial. When the sorbing layers are fully dry (*a*_s_ = 0), an opposite situation is observed. During the first 28 days, water is drawn from the interior, but there is no water loss to the exterior. In fact, the multilayers gain mass through absorption of water from the low activity environs. These results show the importance of controlling the sorbing layer’s water activity in a multilayer design.

#### 4.1.3 Number of Layers

The final simulations investigated the effects of the total number of layers. Simulations were made according to the parameters set in Table 2 where the total barrier thickness was set to 600 μm and the total sorbing layer thickness was set to 400 μm. This total thickness for each material was then evenly distributed across the layers. As the equivalent barrier layer thickness was always set to 600 μm, then any increase in the delay time is due to a better multilayer barrier performance. Figure 13 compares the delay times for each of the multilayers. Increasing the number of layers was found to have a very slight negative effect on the delay time, but it played no other significant effect on the barrier performance. In practice, the layer thickness is somewhat constrained by coating methods. If the layers were of similar thickness but increased in number, the increased overall thickness would positively impact the resulting barrier properties.

**Figure 13:**
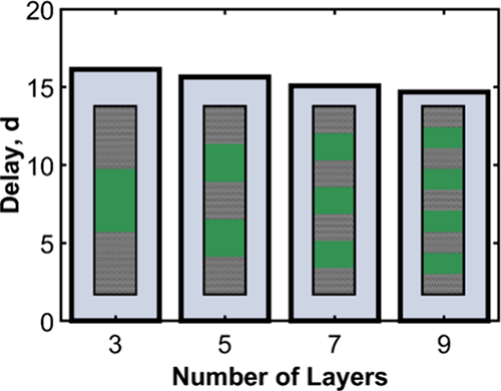
Simulated delay times for three-, five-, seven-, and nine-layer systems (illustrated in the inset diagrams) where the total thickness of the multilayer is held constant.

### 4.2 Experimental Validation

To investigate the validity of the model, three experimental materials systems were devised in increasing practicality as a packaging material. The experimental data of the three trials alongside theoretical predictions are presented below.

#### 4.2.1 Acetate and Glycerine

The initial verification exercise was performed using the model system of a trilayer of 300 μm acetate layers sandwiching 1200 (±2) μm predried glycerine. Glycerine was chosen as it is a liquid and therefore any water absorbed should redistribute on the time scales of the simulations (>15 min). Furthermore, glycerine wetted the acetate and ensured good contact. The experimental results, alongside the model’s prediction, are presented in Figure 14. Whereas there is a constant rate of mass loss through the acetate sheets, there is a mass gain (negative mass loss) when there is a sorbing layer of glycerine. Water from the ambient atmosphere (RH of 53%) was absorbed into the glycerine, leading to the mass gain of the Payne cups. The simulation of the trilayer system was run using the experimental parameters; that is to say, the barrier layers were set to a thickness of 300 μm with a permeability of 0.28 nmol m^–2^ s^–1^. The parameters of the sorbing layer were treated as a fitting parameter and altered (slightly) to match the experimental data. The glycerine was predried at 105 °C for 16 h to remove residual water and then stored in a closed container in an atmosphere with approximately 50% RH. Glycerine is highly hygroscopic and is highly likely to have absorbed water during the storage. Therefore, in the model, *a*_s_ was set to 0.275 (rather than to 0), while the thickness of the glycerine was set to be 1350 μm. A *m*_mono_ value of 30.9, *C* value of 0.857 and a *K* value of 0.972 were obtained by fitting to the sorption isotherm (presented in Figure S6) and were used in the simulations. This thickness resulted in the excellent fit to the data shown in Figure 14, but it is approximately 12% greater than the thickness expected from the mass used in the sample preparation (perhaps because of swelling by water). As is evident from the figure, the model accurately predicts the shape of the mass uptake in the multilayer over a 300-day period. Thus, we conclude that the theoretical model accurately predicts the mass transport in this model system in which the water activity of the sorbing layer is lower than that on both sides of the multilayer.

**Figure 14:**
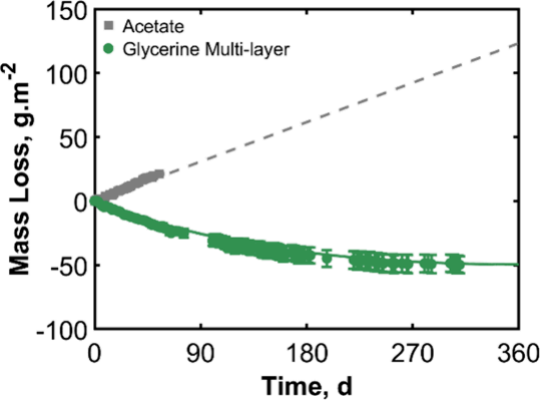
Mass loss over time for stacked acetate sheets and for a trilayer system containing glycerine. The error bars indicate the standard deviation from three replicate measurements. Negative mass loss means that the Payne cup gained mass. Theoretical predictions from our model are presented with a dashed line for the acetate sheets and a solid line for the acetate/glycerine/acetate trilayer barrier system.

#### 4.2.2 Acetate and Chitosan

To make the system closer to practical packaging applications the liquid glycerine was replaced with free-standing chitosan films produced as detailed in the Methods section. Noting the long time scales observed in the acetate/glycerine trilayer, the acetate thickness in this system was reduced to 100 μm. Depositing chitosan films with thicknesses of over 100 μm is impractical due to low solids constraints on the formulation. Free-standing films of chitosan with a thickness of 53 ± 6 μm were found to be reproducible and have a flat surface with minimal defects. These films were stacked in the multilayer efficiently. To increase the amount of chitosan in the multilayers, a five-layer system was considered where two free-standing chitosan films were stacked together to make a sorbing layer of approximately 106 μm. The results are presented in Figure 15. The acetate sheets show a constant rate of mass loss. When the acetate is sandwiching chitosan, there is an initial mass increase, as water is absorbed from the ambient atmosphere. Then, when the chitosan is saturated, there is a mass loss at a rate that is comparable to that of the acetate sheets.

**Figure 15:**
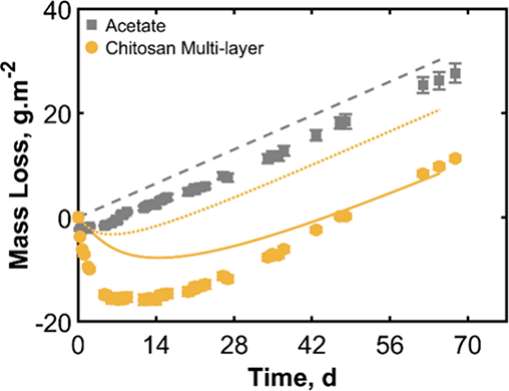
Mass loss over time for stacked acetate sheets and for the five-layer acetate/chitosan multilayer barrier system. The error bars indicate the standard deviation from three replicates. Model predictions are indicated with a dashed line for the acetate and a dotted line for the five-layer acetate/chitosan system. An additional model prediction for the multilayer with thicker chitosan assumed is presented with a solid line.

Model predictions are overlaid on the experimental data. Parameters were selected for the model simulation to ensure a good match to the acetate sheets. A value of 0.19 nmol m^–2^ s^–1^ was used for the acetate permeability as obtained from the experimental data. A thickness of 100 μm was used for the acetate sheets. Two simulations for the chitosan multilayers are presented in Figure 15, both of which used these parameters for the acetate layers. In the first simulation, the chitosan thickness was set to the experimental value of 53 μm, and an *a*_s_ value of 0 was used for the predried chitosan. This simulation is shown with a dotted line. In the second model, the chitosan thickness was instead set to 125 μm (shown as the solid line). The simulation using a 53 μm thickness underpredicts the mass of water that was able to be absorbed by the chitosan, but the delay time appears to be comparable to the experiment. Conversely, when the chitosan sheets were made thicker in the model, the water absorption is more comparable to the experiment, but the delay time is longer. This suggests that the chitosan holds more water than the model assumes. In both cases, the model prediction is on the correct order of magnitude as the experimental result.

These two verification exercises show that the model is able to predict the barrier properties of a multilayer system to the correct magnitude and therefore has some utility in screening barrier designs.

#### 4.2.3 Waterborne Coating and Chitosan Multilayers

Although acetate sheets can be used for some types of packaging, there is also a need for barrier coatings on containers. Hence, we used a waterborne polymeric latex barrier layer deposited on paper with free-standing chitosan sheets as a model system for a practical application. The thickness of waterborne barrier coating was measured (with digital callipers) to be 25 ± 5 μm and the chitosan was 32 ± 10 μm with an *a*_s_ of 0 (predried). The results are presented in Figure 16A.

**Figure 16:**
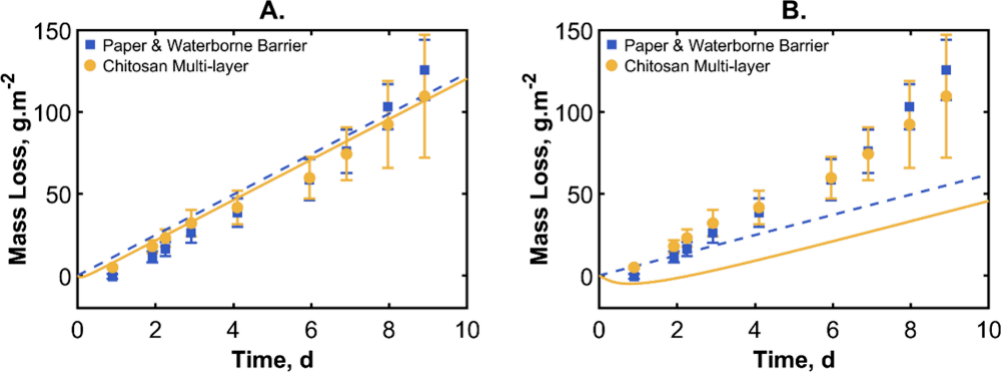
(A) Mass loss over time of two sheets of paper with a waterborne polymeric latex coating and chitosan trilayer barrier system. Error bars indicate the standard deviation of three replicates. Theoretical predictions indicated with a dashed line for the waterborne coating on paper and a dotted line for the chitosan trilayer. (B) Same data as in panel A but the simulation uses more optimal parameters (barrier thickness of 50 μm and sorbing layer thickness of 150 μm) to reduce the water flux temporarily and to introduce a delay time.

The experimental results of the waterborne coating sandwiching the chitosan are nearly identical to those for the coatings without the chitosan. The simulations show a similar result with only a small decrease in the amount of water lost with the addition of the chitosan. We interpret this result as stemming from the relatively high permeability of waterborne coating. An experimental permeability value of 0.85 nmol m^–2^ s^–1^ was used in the simulation. (The permeability of the waterborne coating on paper was determined in preliminary experiments.) The waterborne barrier layer is too poor when at the experimental thickness to obtain any benefit from the addition of the chitosan sorbing layer. We investigated what thicknesses of barrier coating and chitosan layers are needed to see a meaningful delay time and measurable mass uptake. We show in Figure 16B that when increasing the thicknesses of both the chitosan and waterborne coating to 150 μm, there is a benefit from the chitosan layer. On day seven, the multilayered system has lost just 62.5% of the water mass in comparison to the barriers only. Because the chitosan solutions have a high viscosity even with a low solids content, it is difficult to deposit thicker layers. Moreover, the waterborne coatings typically cannot be made thicker than 100 μm while preserving their surface quality. Therefore, there are practical challenges in making a multilayer to achieve some of the predicted barrier properties.

#### 4.2.4 Limitations of the Model and Future Work

The results in the experiments are broadly captured by the model, which suggests that the essential underlying physics is correct. However, our model has some limitations. In real systems, the sorbing layer will have a measurable permeability, and the barrier layer will absorb water that will change the chemical potential and water activity. However, by neglecting the time for diffusion in the sorbing layer, the flux is overpredicted (thus giving a conservative estimate). By not considering water sorption in barrier layers, permanently bound water in the barrier layers is neglected. Thus, the flux is again overpredicted.

Our model assumes that the permeability of the barrier is the same at all points in the plane of the coating. However, if there was a pinhole defect, the permeability would be higher locally. The water flux would then be dominated by the defect and not by the diffusion through the barrier layer. Our model would not be applicable. If there was delamination of the layers in the multilayer structure, the transport mechanisms would potentially change. With an air gap, there would be additional diffusive transport in the air and extra desorption/absorption steps. The model would need to be expanded accordingly.

The model could in future be adapted to consider cyclic humidity conditions, rather than holding the relative humidity constant in the ambient medium. If the ambient RH were to be raised, water might be sorbed into the multilayer, rather flowing out of it. The rate of water flux through the multilayer would be expected to fluctuate in response to the RH changes. This investigation would be relevant to practical situations where packaging is experiencing a range of environmental conditions.

## 5 Conclusions

A theoretical model has been developed to predict the flux of water through a multilayer structure. The key idea is that the differential of water activity across a barrier layer, which is the driving force for water flux, can be temporarily reduced by sorbing water in an adjacent hydrophilic layer. Eventually, after a delay time that can be several days, parity in the water flux is achieved between the multilayer and the equivalent barrier layers without the sorbing layers. Experimental validation experiments showed that the model can accurately predict the trends in the transport of water though the multilayers consisting of water-sorbing material (glycerine and chitosan) sandwiched between acetate sheets, which are good barriers on their own. The benefits of a water-sorbing layer were not apparent in an experiment when the layers are thinner and when the barrier layer originally has a relatively high permeability. In future work, the introduction of crystalline domains^27^ in the barrier and the avoidance of surfactants in the emulsion polymerization^28,29^ could improve the barrier properties of the latex coatings.

Simulations in which key parameters in the model were varied have revealed insights into the optimum designs for multilayer structures including sorbing layers. Specific findings include:

- Improving the barrier layer effectiveness (either by increasing its thickness or decreasing its permeability) in a multilayer structure increases the delay time until the water flux through a multilayer compares to the equivalent barrier. Already good barriers show the greatest benefit of adding hydrophilic layers.
- Distributing the thicknesses of the barriers, such that the first and final layers are thicker than the interior layers, increases the delay time.
- Drying the sorbing layers (to make their initial water activity close to 0) makes them more effective in the multilayer structure by pulling in water from the ambient atmosphere.
- Increasing the water-holding capacity of the sorbing layer (by either increasing the thickness or increasing the *m*_mono_ and *K* values in the GAB isotherm equation) increases the delay time.

Our new model provides a deeper understanding of how to design packaging by counterintuitively including water-sorbing materials. This strategy will allow environmentally sustainable, hydrophilic biomolecules to be applied in packaging of aqueous products and thus to reduce the reliance on petrochemically derived plastics.

## Supplementary Material



## Notes

Data are provided on the effect of delay times on the simulation of mass loss rate; simulations showing the effect of barrier thickness and permeability in a multilayer structure on water mass loss; diagrams of the permutations of multilayer structure used in the simulations; efficiency ratio map with varying GAB parameters; simulations of mass loss rate varying over time for various preconditioning; sorption isotherms with fits to the GAB model (PDF)

## †

Aptitude Medical Systems, 125 Cremona Drive, Goleta, CA, 93117 USA (J.L.K.)
