# Supplementary material for: Modeling and Experiments on Multilayered Barrier Coatings Containing Water-Sorbent Biopolymers
Source: Ind Eng Chem Res. 2025 Nov 25;64(49):23471–85. doi: 10.1021/acs.iecr.5c04116 (PMC12874541; doi:10.1021/acs.iecr.5c04116)
Supplement: Supplementary file 1 [file ie5c04116_si_001.pdf]

Modelling and Experiments on Multi-Layered Barrier Coatings Containing Water-Sorbent Biopolymers

Solomon Stavros Melides[1], Ian Peter Williams[1†], Joseph L. Keddie[1]*

[1]School of Mathematics and Physics, University of Surrey, Guildford GU2 7XH, UK

*Corresponding author. E-mail: j.keddie@surrey.ac.uk

S1. Notes on the Simulations

The rates of water flux in the systems were tested to ensure that the flux through the multi-layers was near equilibrium. The difference between the mass loss of the final two points of the equivalent barrier and the multi-layer were evaluated to determine if parity had been achieved. An effective equilibrium was assumed when the water flux in a multi-layer was found to be ± 5 % the value of the equivalent barrier. If this was not found to be the case, the simulation was run for a longer time frame.

Typically, the simulations were run to predict the mass loss over a 35-day period with δt being 15 minutes. Periods were increased to 95 days (δt = 30 minutes), 180 days (δt = 30 minutes) or 375 days (δt = 60 minutes). The effect of the time intervals was tested to ensure that the intervals did not alter the results. A five-layer system comprising of barrier layers of 100 µm and 1 nmol m^{-1} $s^{-1}$and sorption layers of 2.5 mm and GAB values set to 10, 10 0.85 for the m_{mono}, C and K values, internal a_w of 1 and 0.53 where the sorption layers were set at 0.53 a_w. The model was run with time intervals of 1, 10, 15, 30, 60 and 120 minutes. A comparison of different δt is presented in Figure S1.

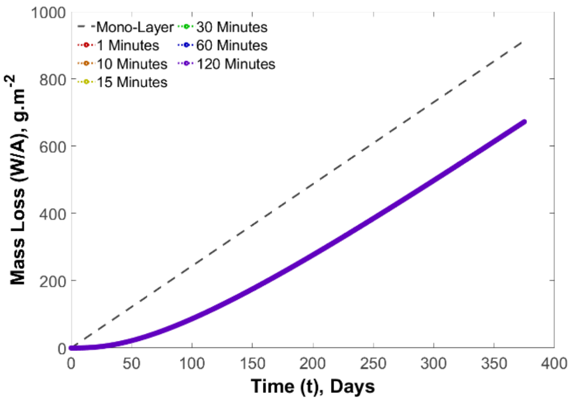

Figure S1. Mass loss of a five-layer system when the time intervals in the simulation were changed from 1 to 120 minutes. The curves fully overlap. The dashed line represents the results for the barrier layers without the addition of sorbing layers.

S2. Checking the Model Predicts the Behaviour of Barrier Layer Equivalents

The model was checked to ensure it functions when predicting the effects of the barrier layer only, without including the sorbing layers. The effects of barrier layer thickness and permeability are presented in **Figure S2**. Results are as expected for the water flux through a single barrier layer.

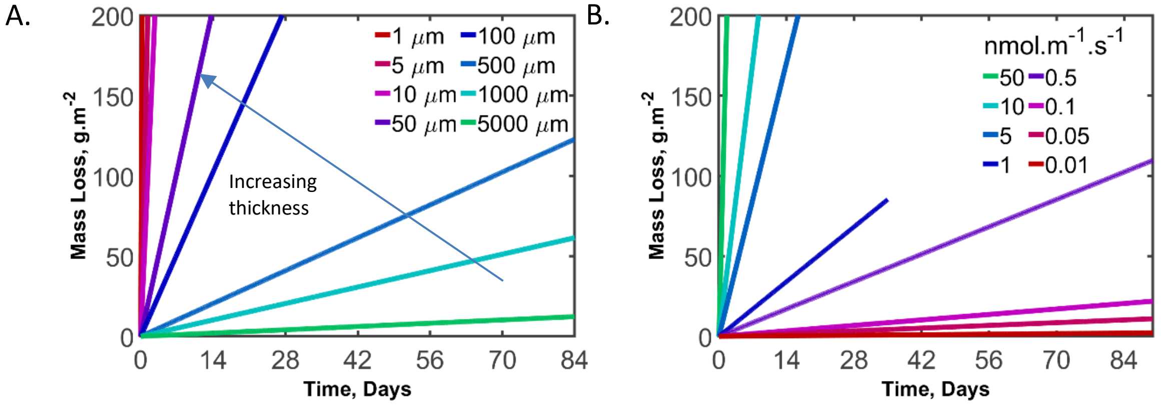

Figure S2. Simulations of the equivalent barrier's water mass loss per unit area obtained from a five-layer system when assuming **A)** various thicknesses of the standard barrier layers with a permeability of 1 nmol m^{-2} s^{-1} and **B)** various permeabilities of the standard layers when the thickness is 100 μm.

S3. Altering the thickness distribution in a five-layer system

Various thickness distributions were assessed by changing the thickness of the three barrier layers and the two sorbing layers in 100 μm increments. The permutations have been organised into three groups based on the thickness distribution of the sorbing layers (red, green and blue) and into a separate 10 groups (α-κ) based on the barrier layer thickness distribution. Other than the thickness, the values used in the model match those presented in **Table 2** on the main article.

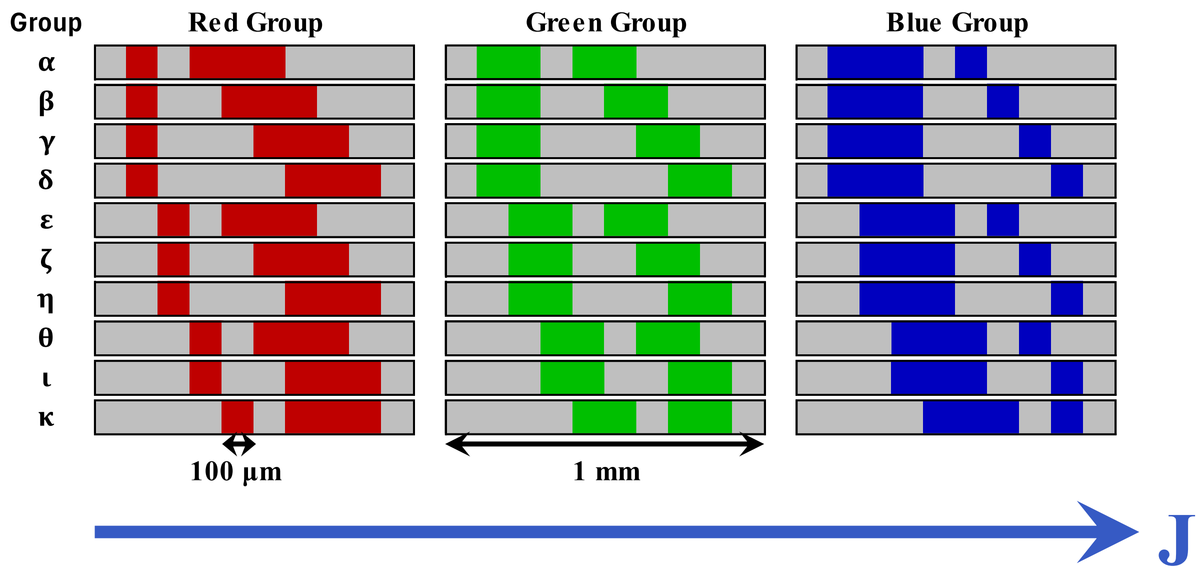

Figure S3. Schematic of the 30 different permutations used to evaluate the effect of thickness distribution in a five-layer system. Schematic diagrams of multi-layer structures. The flux will go from left to right through the five-layer structures. Grey boxes represent the standard barrier layer. The coloured boxes represent the sorbing layers. The red group has permutations where 100 μm of the sorbing layer is closest to the inside of the barrier system (high water activity) and 300 μm toward the outside (low water activity). The green group permutations are evenly distributed sorbing layers (200 μm for each), and the blue group permutations have 300 μm sorbing layers closest to the inside and 100 μm layers toward the outside.

S4. Effect the GAB values on the Efficiency Ratio, r_e

Figure S4 shows the effect of changing the GAB values has on r_e for samples as described in **Table 2** in the main article. A lower value of m_{mono} appears to consistently yield higher efficiency ratios, but the differences between the samples is small. The other GAB values, especially K, have a greater effect on r_e.

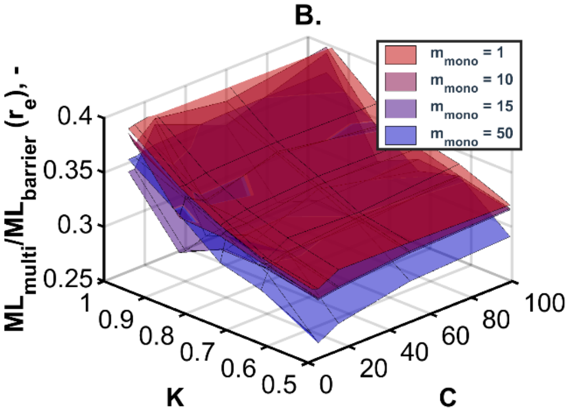

Figure S4. Maps of the efficiency ratio, r_e, (mass loss of the multi-layer prediction as a fraction of the loss from the equivalent barrier) for five-layer systems with the various GAB values in the parameter space.

S5. Water Loss of Various Pre-conditioned Multilayers

The combination of dry and wet pre-conditioned sorbing layers can produce odd-shaped mass loss curves as presented in **Figure S5**. Three examples are given showing how a curve displays a shape indicating either the losing or gaining of mass only for the curve to exhibit the opposite shape as water migrates through the sorbing layers.

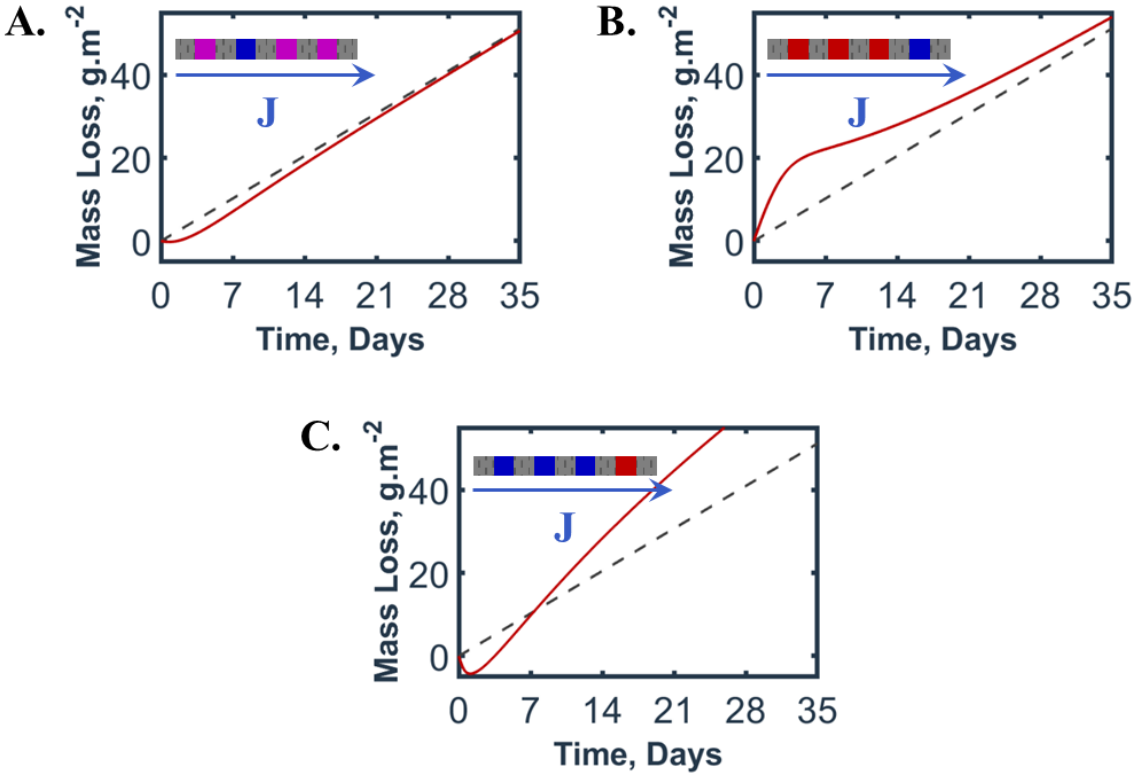

Figure S5. A-C Example mass loss curves where the pre-conditioning gives oddly shaped mass loss profiles. The pre-conditioning is indicated in the schematic in the top left-hand corner. A red box indicates the sorbing layer a_w is set to 0, magenta indicates the sorbing layer is set to an a_w of 0.5, and the blue box indicates the a_w is set to 1. The dashed line shows the simulation for the equivalent barrier layers without a sorbing layer.

S6 Sorption Isotherm of Glycerine and Chitosan

The sorption isotherms for glycerine and chitosan were obtain as detailed in the materials and methods. The results are presented in **Figure S6**. The isotherms were fitted with the GAB equation using MATLAB. The chitosan data was obtained from a free-standing film.

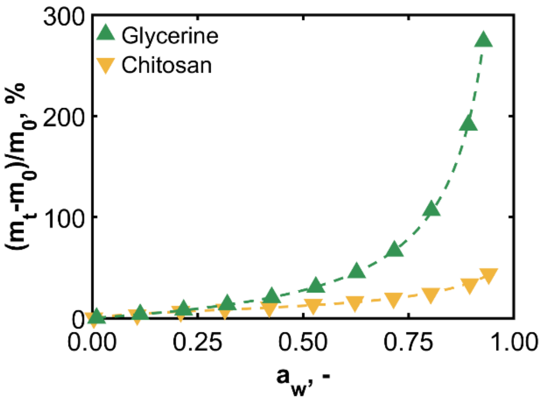

Figure S6. Sorption isotherm of glycerine and chitosan obtained using a dynamic vapor sorption apparatus. The dashed lines indicate the fitting of the GAB equation. For glycerine, the best-fit parameters are: $C = 1.00$; $K = 0.98$; and $m_{mono} = 28.78$. For chitosan, the best-fit parameters are: $C = 8.85$; $K = 0.87$; and $m_{mono} = 7.87$.
